# Descending Thoracic Aortic Diameter Is a Predictor of Poor Outcome After Endovascular Aortic Repair

**DOI:** 10.1093/ejcts/ezag108

**Published:** 2026-02-24

**Authors:** Gabriele Piffaretti, Chiara Lomazzi, Viviana Grassi, Dittmar Böckler, Dennis R Gable, Ross Milner, Gilbert R Upchurch, Santi Trimarchi

**Affiliations:** Vascular Surgery—Department of Medicine and Surgery, University of Insubria Faculty of Medicine and Surgery, Varese 21100, Italy; Vascular Surgery—Department of Cardio-Thoracic and Vascular Surgery, Fondazione IRCCS Cà Granda Ospedale Maggiore Policlinico, Milan, 20122, Italy; Department of Clinical Sciences and Community Health, University of Milan School of Medicine, Milan, 20122, Italy; Vascular Surgery—Department of Cardio-Thoracic and Vascular Surgery, Fondazione IRCCS Cà Granda Ospedale Maggiore Policlinico, Milan, 20122, Italy; Department of Clinical Sciences and Community Health, University of Milan School of Medicine, Milan, 20122, Italy; Vascular and Endovascular Surgery, University Hospital Heidelberg, Heidelberg, 20122, Germany; Vascular Surgery, Baylor Scott & White—The Heart Hospital, Plano, TX, 69120, United States; Vascular Surgery and Endovascular Therapy, University of Chicago Pritzker School of Medicine, Chicago, IL, 75093, United States; Vascular Surgery—Department of Surgery, University of Florida College of Medicine, Gainesville, FL, 60637, United States; Vascular Surgery—Department of Cardio-Thoracic and Vascular Surgery, Fondazione IRCCS Cà Granda Ospedale Maggiore Policlinico, Milan, 20122, Italy; Department of Clinical Sciences and Community Health, University of Milan School of Medicine, Milan, 20122, Italy

**Keywords:** TEVAR, thoracic endovascular aortic repair, giant aneurysm, freedom from reintervention, aorta-related mortality, endograft infection, open conversion

## Abstract

**Objectives:**

To evaluate the results of thoracic endovascular aortic repair (TEVAR) in large (diameter ≥7 cm) aneurysms of the descending thoracic aorta.

**Methods:**

This cohort has been extrapolated from the prospective, observational (on-label and off-label), worldwide multicentre Global Registry for Endovascular Aortic Treatment (GREAT) (NCT01658787). Patients were divided into 2 groups based on the baseline aortic diameter: standard aneurysms (<7 cm) and larger aneurysms (≥7 cm). Primary outcomes were overall survival and freedom from TEVAR-related reintervention. Secondary outcomes were freedom from aortic-related mortality (ARM), as well as from type 1 endoleaks, and cumulative risk of TEVAR-related infection and/or aorto-bronchial/oesophageal fistulization.

**Results:**

We analysed 613 (80.4%) patients with standard aneurysms and 149 (19.6%) with larger aneurysms. Demographic data and comorbidities were not different between the groups. At the 4- to 6-year window, 496 (65.1%) patients remained under follow-up (standard, *n* = 409 [66.7%] vs large, *n* = 87 [58.4%]; odds ratio [OR]: 1.4, *P* = .056). Large aneurysm diameter was independently associated with higher hazards for all-cause mortality (hazard ratio [HR]: 1.6, 95% CI, 1.19-2.20; *P* < .001), TEVAR-related reintervention (HR: 2.4, 95% CI, 1.52-3.65; *P* < .001), risk of ARM (HR: 2.2, 95% CI, 1.03-4.75; *P* = .026), cumulative risk of TEVAR-related infection/fistulization, and type 1 endoleaks (HR: 3.3, 95% CI, 1.89-5.65; *P* < .001).

**Conclusions:**

Preoperative descending thoracic aortic diameter seems to be an important determinant of outcomes after TEVAR, where patients presenting with aortic diameter ≤ 7 cm showed more favourable long-term outcomes.

## Graphical abstract

**Figure:**
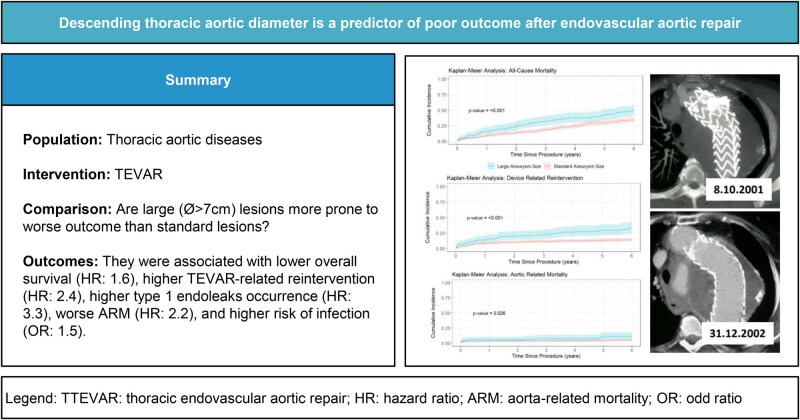

## Introduction

Although studies have shown that maximum diameter does not fully predict aneurysm-related complications, currently it remains the most commonly used criterion determining the indication for intervention in aortic aneurysms.^1–4^ Size-dependent risk has been demonstrated across different aortic segments. In previous abdominal aortic aneurysms analyses, outcomes following endovascular repair are significantly worse when diameter exceeds 6 cm.^5–8^ Similarly, for the descending aorta, an increased rate of complications has been reported when the diameter exceeds 7 cm.^9^ Contemporary real-world data indicate that thoracic endovascular aortic repair (TEVAR) is associated with improved early outcomes compared with open surgical repair (OSR).^10–12^ The favourable results are most evident in smaller and less extensive aneurysms, which are anatomically better suited to the minimally invasive nature of TEVAR.^13^ Moreover, large thoracic aneurysms are relatively uncommon and have therefore rarely been analysed as a distinct population after TEVAR: as a consequence, TEVAR outcomes stratified by aortic diameter remain scarce and has limited the ability to evaluate long-term outcomes in this subgroup.^14–21^ Therefore, the aim of this study was to assess the long-term outcomes of TEVAR in patients with large thoracic aortic aneurysms using data from a worldwide multicentre registry.

## Methods

### Ethical statement

This study was conducted according to the Declaration of Helsinki and the International Conference on Harmonization and Good Clinical Practice guidelines and approved by the ethical committee or institutional review board of each participating centre (protocol n. 0038114, November 6, 2013). Written informed consent was obtained from all participants. Data were acquired and then handled to the authors by the sponsor.

### Patients’ cohort

**Figure 1.**
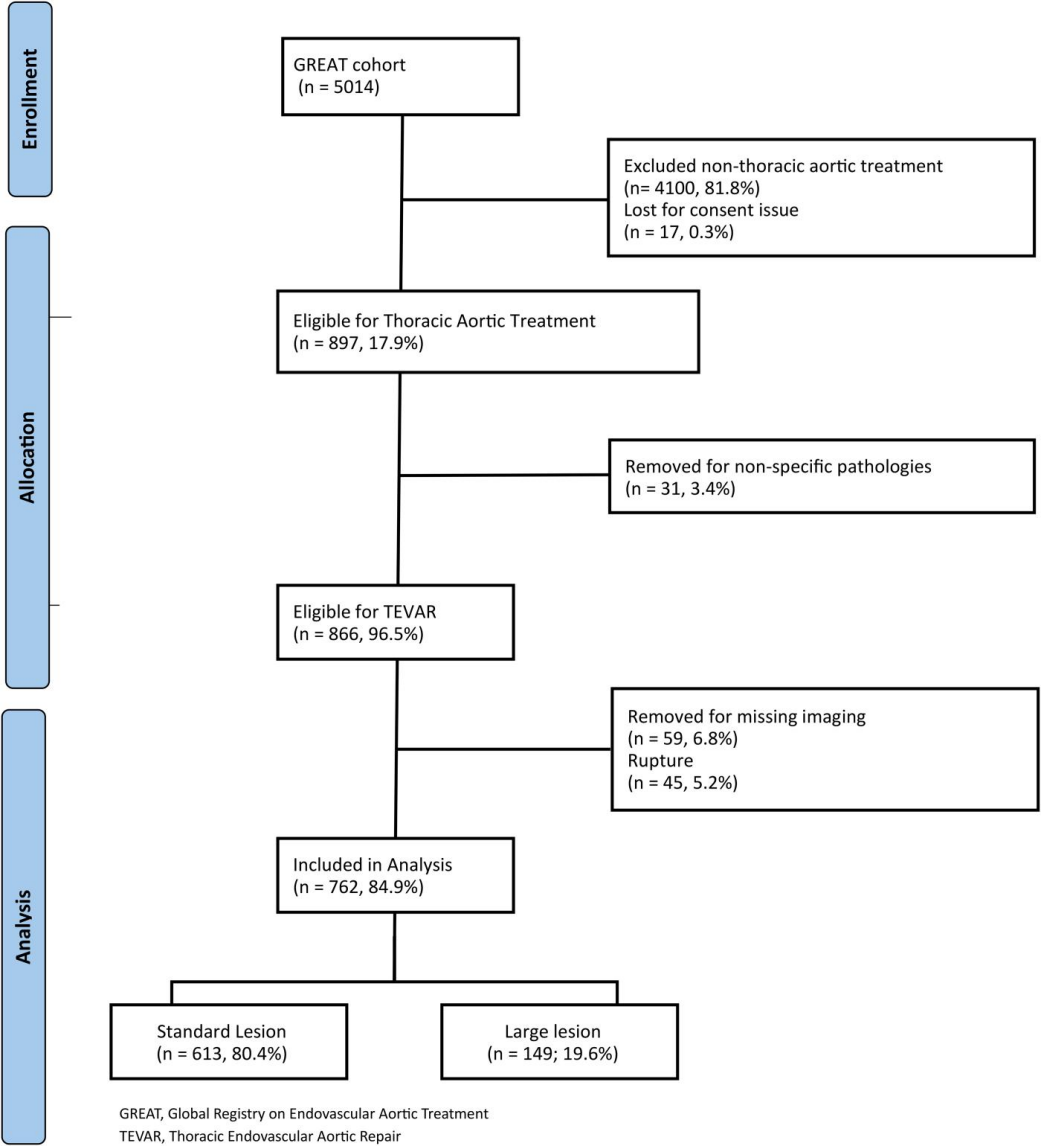
Consort Diagram of the Whole Aortic Cohort Enrolled in the GREAT Registry. Abbreviations: GREAT, Global Registry on Endovascular Aortic Treatment; TEVAR, thoracic endovascular aortic repair.

This study cohort was extrapolated from patients enrolled in the Global Registry for Endovascular Aortic Treatment (GREAT), a prospective, observational, multicentre registry that includes both on-label and off-label use of endovascular devices and is registered with ClinicalTrials.gov (NCT01658787). The GREAT Registry is a sponsored registry rather than a privately operated programme. Oversight has included review by an independent advisory committee throughout the entire study period, which extends nearly 10 years. In addition to this governance structure, risk-based monitoring has been applied to all collected data. This monitoring was performed by an independent contracted research organization assigned to each participating region or site, with frequency and scope determined by patient enrollment intervals. Patients were consecutively enrolled at each participating centre by the treating physician in accordance with predefined eligibility criteria and at the time of endovascular treatment; data collection was conducted in accordance with the registry protocol. The GREAT inclusion criteria are limited to enhancing patient enrollment that reflects real-world practice. Inclusion criteria consisted of any minimum age requirement by state or country regulations (typically 18 years old or older), an indication for an aortic endograft (EG) repair as determined by the treating physician, and a signed informed consent. The GREAT received Institutional Review Board or Ethics Committee approval at each study site. Off-label use, non-standard indications, and devices deployed outside instruction for use (IFU) have been included in the GREAT. Exclusion criteria include patients who do not have informed consent or do not meet any minimum age requirement by state or country’s minimum regulation. Specific details regarding the registry design, participating centres, recruitment period, and patient inclusion process have been previously described by Loa et al and subsequent publications.^14^^,^^22^^,^^23^ With respect to device selection, the registry was intentionally designed as a priori as an industry sponsored initiative focused exclusively on patients treated with W.L. Gore & Associates aortic devices. The objective of the registry was to evaluate the real-world outcomes and performance of W.L. Gore & Associates devices rather than to compare devices across manufacturers. Consequently, patients treated with alternative EGs are not reported here. Such device comparisons were beyond the scope of this registry. The registry design anticipated longitudinal evaluation of device-related outcomes across a broad range of aortic pathologies, consistent with standard expectations for post-market evidence generation in endovascular therapy. The primary and secondary end-points outlined in the original programme description demonstrate a clear intention to support analyses of this nature, including pathology-specific outcomes and long-term device performance. Enrollment consisted of consecutive patients treated with W.L. Gore & Associates aortic devices at participating centres, and data collection adhered to prespecified registry procedures. Collected data included demographics, comorbidities, morphologic characteristics of the aortic aneurysms, case planning details, type of intervention and EG used, as well as follow-up outcomes, including serious postoperative adverse events such as complications, death, and need of aorta-related reintervention. For this specific analysis, inclusion criteria comprised patients with intact aortic aneurysms, including degenerative atherosclerotic aneurysms, penetrating aortic ulcers, and type B dissection-related aneurysms. Exclusion criteria included rupture, intramural haematoma, ascending aorta diseases, rare aortic conditions such as Kommerel diverticulum, floating thromboatheroma, aorto-bronchial or oesophageal fistula, coarctation, acute traumatic injury, and absence of informed consent. A post-hoc analysis classified patients into 2 groups based on baseline aortic aneurysm diameter: standard (<7 cm) and large (≥7 cm), as shown in **Figure 1**.

### Registry-based definitions and major outcomes

A universally accepted anatomic definition of a large thoracic aortic aneurysm has not been established: therefore, specifically for the purposes of this study, a large aneurysm was defined as a transverse aortic diameter of at least 7 cm, consistent with the most recent guideline recommendations.^2^ Follow-up surveillance typically consisted of computed tomography angiography (CT-A) or magnetic resonance imaging at 1 month, 12 months, and annually thereafter, with additional imaging performed as clinically indicated. The GREAT study protocol did not mandate fixed intervals for follow-up visits or imaging, which were determined at the discretion of the treating physician. TEVAR-related reinterventions were defined as any invasive or minimally invasive procedures performed in relation to the initial repair. Specifically, these included any procedures undertaken to address deficiencies of failures of the endovascular devices(s) implanted in the aorta at any time after the initial intervention. Specifically for the purposes of this study, the primary outcomes were all-cause mortality, and freedom from TEVAR-related reintervention. Secondary outcomes included freedom from aortic-related mortality (ARM), and freedom from type 1 endoleaks. In addition, the cumulative incidence of TEVAR-related infection and/or aorto-bronchial/oesophageal fistulization was evaluated. Participating centres were required to report only adverse events that met the International Organization of Standardization definition of serious adverse event, as specified in ISO 14155 (https://www.iso.org/obp/ui/#iso: std: iso : 14155: ed-2: v1: en).

### Statistical analysis

Collected data were recorded on a web-based electronic report form (iMedidata, Medidata Worldwide Solutions, Inc., New York, NY, USA) to ensure reliability and secure authentication and traceability. Data management was performed by the Gore Clinical Research Department (W.L. Gore & Associates). All collected data were reviewed, and if missing or inconsistent data were detected, relevant queries were posed to the investigators for resolution. Consistence between electronically imported data and source documents was also examined. Categorical variables were summarized as counts and percentages, and continuous variables as mean ± standard deviation (SD) or median with interquartile range (IQR). Count variables were analysed via Fishers’ Exact test. Time-to-event outcomes (ARM and freedom from TEVAR-related reintervention) were estimated with Kaplan-Meier methods utilizing log-rank *P*-value calculations. Cox proportional hazards models were used for univariable and multivariable analyses, with censoring at 2 years for participants without an event. Univariable models include demographic (age, race, gender, weight, medical history) and anatomical/procedural (aneurysm diameter, proximal neck length, branch vessel procedure, access vessel technique and site) covariates. Proportional hazards assumptions were tested by adding time interaction terms. Interactions with *P* ≤ .10 were retained. Multivariable models were constructed using backward selection to maintain numerical stability. All *P*-values were 2-sided with significance defined as < .05. Analyses were conducted using R version 4.5.1 for Windows.^24^

## Results

### Study population

Out of the 5014 patients enrolled in the registry, 762 (15.2%) met the eligibility criteria for this analysis, including 613 patients (80.4%) with standard aneurysms and 149 patients (19.6%) with larger aneurysms. No significant differences in baseline demographic characteristics or comorbidities were observed between the 2 cohorts, as shown in **Table 1**. In contrast, the distribution of underlying aortic pathology differed between the groups, as detailed in **Table 2**.

**Table 1.** Demographic Data, Comorbidities, and Risk Factors of the 2 Cohorts

Covariates	Small aneurysms	Large aneurysms	OR	*P*
	(*n* = 613)	(*n* = 149)		
*Demographics, (%)*				
M: F	402:211	105:44	0.8	.257
Age, mean (DS)	67 (12)	68 (11)		.354
*Comorbidities, (%)*				
Hypertension	542 (88.6)	128 (85.9)	1.3	.367
Smoking habit	341 (55.7)	77 (51.7)	1.2	.385
Hypercholesterolemia	294 (49.5)	76 (53.9)	0.8	.351
Coronary artery disease	162 (26.8)	44 (29.9)	0.9	.495
Chronic obstructive pulmonary disease	142 (23.4)	33 (22.1)	1.1	.759
Cardiac arrhythmia	113 (18.5)	35 (23.5)	0.8	.397
Diabetes	84 (13.8)	27 (18.1)	0.7	.190
Peripheral arterial obstructive disease	82 (13.5)	20 (13.6)	1.0	.913
Congestive heart failure	51 (8.3)	18 (12.2)	0.7	.154
Stroke	47 (7.7)	14 (9.5)	0.8	.486
End-stage renal disease	15 (2.5)	6 (4.1)	0.6	.296
Connective tissue disorder	15 (2.5)	6 (4.1)	0.6	.296
*Risk factors*				
BMI, median (IQR)	27.5 (13.6-63.8)	27 (16.7-54.6)		.711
Outside IFU (≥1 feature)	338 (55.1)	99 (66.4)	1.6	.013

Abbreviations: BMI, body mass index; DS, deviation standard; IFU, instruction for use; IQR, interquartile range; n, number; OR, odds ratio.

**Table 2.** Aortic Disease Classification

Covariates	Small aneurysms	Large aneurysms	OR	*P*
	(*n* = 613)	(*n* = 149)		
*Aortic pathology, (%)*			0.4	< .001
Atherosclerotic	405 (66.1)	123 (82.5)		
Dissection-related	208 (33.9)	26 (17.5)		
Complicated	116	15		
Uncomplicated	92	11		
*Aortic extent, (%)*			2.7	< .001
Aortic arch	18 (2.9)	12 (8.1)		
Descending thoracic	535 (87.3)	107 (71.8)		
Throraco-abdominal	60 (9.8)	30 (20.1)		

Abbreviations: OR, odds ratio; n, number.

### Procedural details

TEVAR was performed as the index procedure in 650 (85.3%) patients, whereas 112 patients (14.7%) had undergone prior endovascular repair or OSR. TEVAR was more frequently performed as the index procedure in patients with standard aneurysms (odds ratio [OR]: 2.4, *P* < .001). Femoral artery access was used in the majority of cases (*n* = 692, 90.8%), while a conduit (surgical, *n* = 54; endovascular, *n* = 16) was used in 70 (9.2%) cases, with no difference between the 2 groups (OR = 0.7; *P* = .446). Overall, 350 (45.9%) patients were treated using a fully percutaneous approach. Endograft deployment occurred in proximal landing “zone 0” in 14 (1.8%) patients, in “zones 1 or 2” in 176 (23.1%) patients, and in “zones 3 or 4” in 381 (50.0%) patients. The distribution of proximal landing zones requiring supra-aortic vessel debranching (“zones 0-to-2”) did not differ between groups (*n* = 159 [25.9%] vs *n* = 31 [20.8%]; OR: 1.3, *P* = .195). Based on the device manufacturers’ indications, 437 (57.3%) patients were treated outside the IFU: 338 (55.1%) in the standard group, and 99 (66.4%) in the large group (OR: 0.6, *P* = .013).

### Long-term results (≥ 365 days)

**Figure 2.**
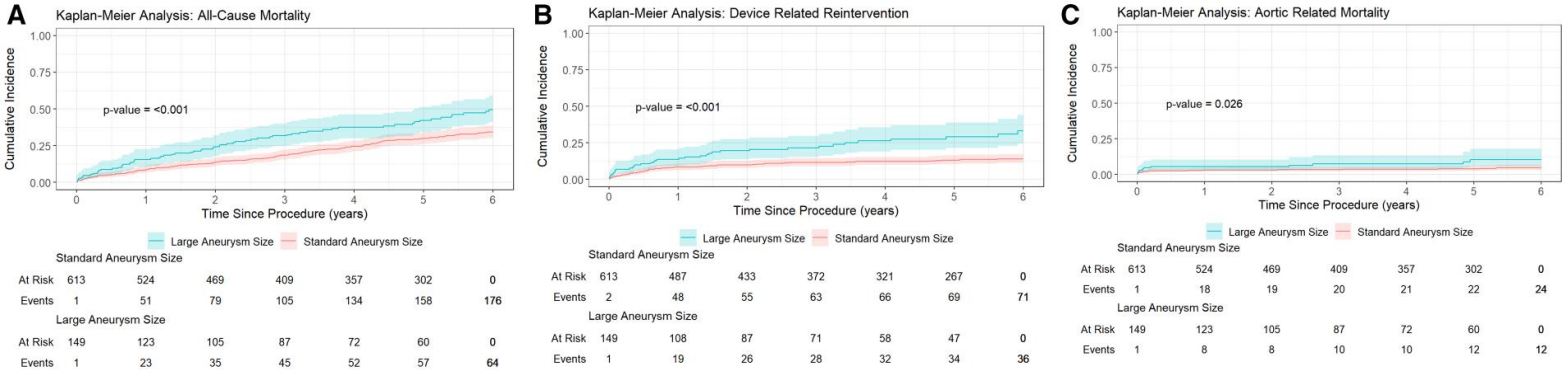
Kaplan-Meier estimates of all-cause mortality (A), TEVAR-related reintervention (B), and freedom from aorta-related mortality (C).

At the 2- to 3-year follow-up window, data were available for 647 (84.9%) patients (standard, *n* = 524 [85.5%] vs large, 123 [82.5%]; OR: 1.2, *P* = .371). At the 4- to 6-year follow-up window, 496 (65.1%) patients remained under follow-up (standard, *n* = 409 [66.7%] vs large, *n* = 87 [58.4%]; OR: 1.4, *P* = .056). Specifically for this analysis, multivariable Cox proportional hazard models were adjusted for age and gender: large aneurysm size was independently associated with higher hazards for all-cause mortality (hazard ratio [HR]: 1.6, 95% CI, 1.19-2.20; *P* < .001; **Figure 2A**) and TEVAR-related reintervention (HR: 2.4, 95% CI, 1.52-3.65; *P* < .001; **Figure 2B**). In addition, the risk of ARM was higher among patients with larger aneurysms (HR: 2.2, 95% CI, 1.03-4.75; *P* = .026; **Figure 2C**). Age only was associated with all-cause mortality, with an approximately 5% increase in risk per year (HR 1.05, 95% CI, 1.04-1.06; *P* < .001) and was not associated with the other outcomes. Overall, GREAT captured 11 (1.4%) cases of TEVAR-related infections (standard, *n* = 5 [0.8%] vs large, *n* = 6 [4.0%]; OR: 0.2, *P* = .008) and 7 cases of fistulization (standard, *n* = 6 [0.9%] vs large, *n* = 1 [0.7%]; OR: 1.5, *P* = .726). Consequently, the cumulative risk of TEVAR-related infection and/or fistulization was higher in the large group (1.8% vs 4.7%; OR: 0.4, *P* = .044).

### Longitudinal reporting on morphological outcome

Overall, mean aneurysm diameter was smaller in the standard group than in the large group (mm, 46.8 ± 12.8 vs 65.0 ± 19.1; *P* < .001). Over the entire follow-up period, aneurysm diameter decreased in both groups, with a greater absolute reduction observed in the large aneurysm group compared with the standard group (mm, −7.4 vs −3.1; *P* = .066). At the last available follow-up, there were no group differences in sac shrinkage (51.2% vs 58.6%; OR: 0.7, *P* = .469), sac stability (35.0% vs 24.1%; OR: 1.6, *P* = .286), or sac enlargement (13.7% vs 17.2%; OR: 0.8, *P* = .650). Large aneurysm size was independently associated with a higher hazard for any endoleak (HR: 2.6, 95% CI, 1.68-4.04; *P* < .001). Freedom from type 1 endoleaks was lower in large aneurysm group (HR: 3.3, 95% CI, 1.89-5.65; *P* < .001). No type 3 endoleak occurred in the larger aneurysm group (0% vs 0.8%; OR: 0.4, *P* = .517). Type 4 endoleak, device migration, fracture, and compression were not observed in either group.

## Discussion

The main finding of this analysis is that large aortic aneurysms (≥7 cm in diameter) are associated with worse major aortic-related outcomes compared with smaller aneurysms (<7 cm in diameter). We acknowledge that the use of a 7 cm threshold to define large aneurysms is inherently arbitrary and may not represent an optimal or universally accepted cutoff. Nevertheless, although more recent data suggest an exponential increase in the risk of adverse events beginning at diameters of approximately 6 cm, our choice aligns with evidence from the traditional literature demonstrating that the probability of adverse events increases markedly at a tipping point around 7 cm.^2^^,^^4^^,^^9^^,^^19^^,^^25^

Recent cardiovascular societies guidelines have emphasized that high-quality registry-based studies are equally important for monitoring changes in clinical practice and outcomes. Defining the target population represents a critical first step in ensuring the reproducibility and interpretability of a registry-based analysis. The low prevalence of large aortic aneurysms reported in the literature may be explained by several factors, including the well-established association between increasing aortic diameter and rupture risk, as well as aggressive surveillance strategies and consensus guidelines recommending operative repair once aneurysm size reaches thresholds at or below 6 cm. The GREAT registry provides a substantial opportunity for robust data analysis because its global scope enables the inclusion of patients from multiple countries and geographic regions, thereby enhancing the generalizability of the findings to real-world practice. Notably, in the present study, the incidence of large aortic aneurysm was not negligible, approaching 21%, which is comparable to the 24% reported by Gallitto et al^19^ in a recent multicentre analysis of endovascular repair for thoraco-abdominal aortic aneurysms.

Success in aortic surgery is traditionally defined as complete exclusion or removal of the aneurysm with the absence of complications. Although TEVAR does not permit aneurysm excision, it has long been considered the benchmark alternative to OSR and, as such, should be evaluated using compatible outcome measures. In a single-centre experience, Giles et al^18^ reported that following TEVAR for acute and chronic type B dissection larger initial aortic diameter was strongly associated with the need for secondary aortic interventions. In our analysis, thoracic aortic diameter was strongly associated not only with the need for TEVAR-related reintervention but also with overall survival. Although findings in the literature regarding the latter outcome are inconsistent, the association between larger aneurysm size and poorer survival may be partially explained by the increased incidence of ARM observed in patients with large aneurysms. Data specifically addressing this relationship in the context of TEVAR remain limited. However, our findings are supported by evidence from the abdominal aortic aneurysm literature. In particular, a systematic review and meta-analysis by Khashram et al^8^ demonstrated that larger aortic diameter independently influences late overall survival, with a stronger association observed among patients treated with endovascular repair.

One of the principal criticisms of endovascular aortic repair is its higher rate of technical complications compared with OSR, and TEVAR is not exempt from this concern. Sac reperfusion, particularly type 1 endoleak, has been consistently reported as the most frequent complication during follow-up and the leading indication for reintervention.^2^^,^^11^^,^^14^^,^^18^^,^^19^^,^^25^ Our analysis corroborates these observations, demonstrating that larger aneurysms developed endoleaks more frequently and, notably, have a higher incidence of type 1 endoleaks. Although the GREAT registry was not designed to capture specific complications or adjudicated individual outcomes, another potentially life-threatening event that may be reasonably associated with persistent aneurysm sac pressurization fistulization. Data from the European Registry of Endovascular Aortic Repair Complications reported by Czerny et al^16^ showed that the median aneurysm sac diameter among patients who developed bronchial or oesophageal fistulas exceeded 9 cm. In the present analysis of the GREAT registry, we observed a markedly higher risk of infection and/or fistulization in patients with large thoracic aortic aneurysms. This increased risk may be related to adjacent organ injury caused by extensive mediastinal thrombohaematoma formation, which can result in compression of the oesophagus or the bronchial tree.

When these unfavourable long-term clinical and technical outcomes are considered alongside the occurrence of highly fatal infectious complications, our findings raise the question of whether preoperative aortic diameter should be given greater weight in guiding the selection of primary OSR among patients with large aneurysms who are otherwise suitable surgical candidates.^26^

### Limitation

The present analysis has several important limitations. First, the non-randomized design of the registry introduces the potential for incomplete data and heterogeneity in patient selection and procedural planning, as management decisions were left to the discretion of the treating physician. Moreover, although data collection was prospective, the analysis was retrospective in nature. The 2 cohorts were not matched, particularly with respect to underlying aortic pathology and the use of EGs outside the IFU. As a result, the inclusion of off-label procedures may limit the reproducibility of the findings. Second, a degree of sample size bias is present, as the analysis was restricted to pathologies typically associated with aneurysmal development. In addition, the choice of a 7 cm cutoff to define large aneurysms, although supported by portions of the existing literature, remains arbitrary and may not represent an optimal or universally accepted threshold. Third, the use of propensity score matching could have strengthened the analysis by better accounting for confounding factors and by helping to distinguish association from causation, particularly given the possibility that selection bias rather than aneurysm size alone may have influenced outcomes. However, implantation of such an approach was not feasible because treatment outside IFU does not necessarily coincide with off-label use, and there was substantial variability in both the number and type of devices employed outside the approved indications. Fourth, the GREAT registry does not mandate the collection of reference imaging for centralized review, precluding detailed analyses of preoperative and postoperative aortic morphology. Consequently, potentially relevant factors such as thoracic aortic anatomy, the distribution of thrombus/atheroma, and thoracic tortuosity at the time of intervention could not be evaluated. Finally, only a limited number of patients had follow-up extending to 6 years, in part because follow-up schedules and reintervention protocols varied across participating centres. Despite these limitations, which may restrict the generalizability of the results, our findings are consistent with the available literature. Periodic audits conducted by independent agencies ensured the reliability of the collected data. In addition, the proportion of patients with available clinical and imaging follow-up at the 6-year time point was acceptable given the observational nature of the study. To our knowledge, no previous long-term analyses have reported outcomes stratified by standard versus large aneurysm size in this manner. Most importantly, the present study provides a meaningful comparative assessment in an area where the literature lacks detailed operative risk stratification based on aneurysm diameter.

## Conclusion

In patients with descending thoracic aortic disease, preoperative thoracic aortic diameter appears to be an important determinant of both clinical and technical outcomes after TEVAR. Patients presenting with an aortic diameter ≤ 7 cm demonstrated more favourable long-term results. Although prospective evaluation of larger cohorts is warranted, preoperative thoracic aortic diameter may represent a relevant parameter in guiding the selection of OSR rather than TEVAR in medically fit candidates.

## Data Availability

The data underlying this article will be shared at reasonable request to the corresponding company.
